# Mutant collagen *COL11A1* enhances cancerous invasion

**DOI:** 10.1038/s41388-021-02013-y

**Published:** 2021-09-28

**Authors:** Carolyn S. Lee, Zurab Siprashvili, Angela Mah, Tomas Bencomo, Lara E. Elcavage, Yonglu Che, Rajani M. Shenoy, Sumaira Z. Aasi, Paul A. Khavari

**Affiliations:** 1 Stanford Program in Epithelial Biology, Stanford University, Stanford, CA 94305 USA; 2 Stanford Cancer Institute, Stanford University, Stanford, CA 94305 USA; 3 Veterans Affairs Palo Alto Healthcare System, Palo Alto, CA 94304 USA

**Keywords:** Cancer, Genetics

## Abstract

Collagens are the most abundant proteins in the body and comprise the basement membranes and stroma through which cancerous invasion occurs; however, a pro-neoplastic function for mutant collagens is undefined. Here we identify *COL11A1* mutations in 66 of 100 cutaneous squamous cell carcinomas (cSCCs), the second most common U.S. cancer, concentrated in a triple helical region known to produce trans-dominant collagens. Analysis of *COL11A1* and other collagen genes found that they are mutated across common epithelial malignancies. Knockout of mutant *COL11A1* impairs cSCC tumorigenesis in vivo. Compared to otherwise genetically identical *COL11A1* wild-type tissue, gene-edited mutant *COL11A1* skin is characterized by induction of β1 integrin targets and accelerated neoplastic invasion. In mosaic tissue, mutant *COL11A1* cells enhanced invasion by neighboring wild-type cells. These results suggest that specific collagens are commonly mutated in cancer and that mutant collagens may accelerate this process.

## Introduction

Cancers of the epithelial tissues that line body surfaces account for more than 90% of human malignancies and the vast majority of cancer mortality [1]. Arising in tissues, such as the skin, lung, prostate, colon, and breast, biologic malignancy of epithelial neoplasms requires neoplastic cells to invade through their underlying epithelial basement membranes into surrounding stromal tissue [2]. Understanding the mechanisms that enable local invasion in epithelial cancer progression may identify targets to interrupt the process before it leads to the negative cancer health impacts of local tissue destruction and distant metastases. The extracellular environment provides cues to enable local cancer invasion, however, the pro-invasion factors secreted from cancer cells themselves are still being characterized.

The collagen gene family [3, 4] encodes fibrillar and non-fibrillar collagen components of both basement membranes and of the extracellular stromal matrix through which cancer cells must penetrate during local neoplastic invasion. Collagens have thus been primarily appreciated for their roles in sustaining normal tissue structure as well as their functions as passive barriers to cancer cell invasion, although recent data support a role in regulating tumor immunity [5]. A number of enzymes can modify collagen proteins, including matrix metalloproteinases (MMPs) [6], which can cleave collagens to disrupt collagen matrix networks. MMP cleavage of collagens has been extensively studied in neoplasia, with a focus on efforts to develop therapeutics that inhibit this process [7]. Mutations in collagen genes themselves, however, have not been widely noted in cancer. An exception to this is *COL2A1*, which was observed in one study to be mutated in 37% of spontaneous human chondrosarcomas [8]. The biologic significance of *COL2A1* mutations in this setting, however, is unknown. The collagen mutations evident in databases, such as those of the cancer genome atlas (TCGA), have been largely overlooked, perhaps because collagen proteins are encoded by large genes that may be susceptible to accumulating background mutations during carcinogenesis.

Among collagen genes, *COL11A1* is of notable interest in cancer [9]. *COL11A1* encodes the type XI collagen α1 chain, a minor fibrillar collagen whose major known action is to regulate the diameter of other major collagen protein fibrils [10]. *COL11A1* overexpression has been associated with poorer progression-free survival in multiple studies [11]. The association of *COL11A1* overexpression with decreased cancer survival spans a diverse array of neoplasms, including head and neck, breast, ovarian, colon, and lung, suggesting a potentially broad role for *COL11A1* in a wide spectrum of cancers [9, 12–14]. Further supporting a possible role for COL11A1 in cancer are observational data linking it to angiogenesis, drug resistance and metastasis [9]. Finally, COL11A1 is a protein binding partner of COL2A1, noted above, suggesting that disruption of the COL11A1–COL2A1-containing heterotrimer could play potential a role in cancer. Mutant *COL11A1*, however, has not been characterized in cancer and a functional role for the COL11A1 protein in promoting neoplastic progression to cancerous invasion in tissue has not been studied.

Cutaneous squamous cell carcinoma (cSCC) has a U.S. annual incidence estimated as high as 2.7 million [15] and may cause comparable numbers of deaths to malignant melanoma [16]. cSCC commonly arises in sun-exposed skin and progresses from premalignant actinic keratoses and cSCC in situ to locally invasive cancer by invasion through the epidermal basement membrane. cSCC is thus a prototype of epithelial malignancy, which progresses through discrete local stages prior to metastasis. The latter occurs in ~4% of cSCC patients, with roughly a third of metastatic cSCC leading to death [17]. Because of the accessibility and tractability of skin to organoid formation and grafting, human skin tissue has been amenable to production of a variety of cancer models that accurately reflect the tissue architecture and gene expression observed in spontaneous cSCC arising in humans [18–20]. A number of mutations have been reported in cSCC studies, with *TP53* the most commonly mutated gene noted [21]. A number of other genes implicated in cancer pathogenesis have also been observed to be recurrently mutated in human cSCC, such as *NOTCH1*, *CDKN2A*, *KNSTRN*, and *HRAS* [22–25]. cSCC, however, was not a focus of TCGA efforts and its mutational spectrum has not been fully characterized.

To begin to address this, we analyzed the mutational spectrum of 100 cSCC exomes as well as 100 patient- and site-matched normal control skin samples, including 67 previously published tissue pairs and 33 new for this study. Surprisingly, *COL11A1* was identified among the top recurrently mutated genes in cSCC. *COL11A1* point mutations were found to both substantially boost local neoplastic invasion in vivo as well as to be required for subcutaneous tumorigenic growth in cSCC models. Mutant *COL11A1*-activated β1 integrin targets and accelerated neoplastic invasion non-cell autonomously. In TCGA data, collagen gene mutations were found widely across other common epithelial cancers. Rather than predominantly functioning as passive structural conduits for cancer invasion, therefore, collagens are also frequently mutated in epithelial cancers where they can accelerate neoplastic progression.

## Results

### Somatic *COL11A1* mutations in cSCC and other human cancers

**Fig. 1:**
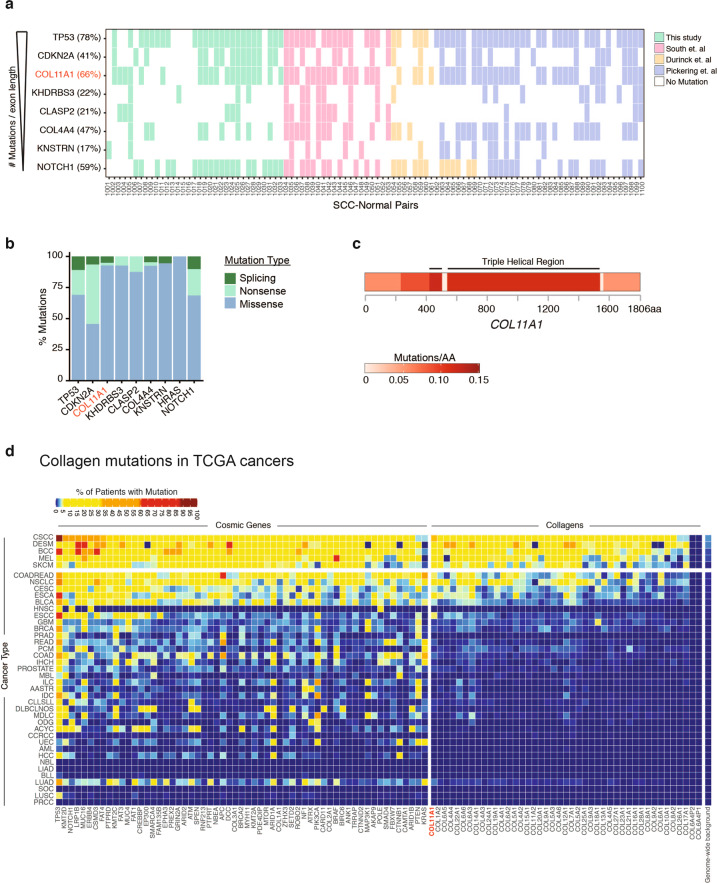
*COL11A1* is frequently mutated in cSCC and other human cancers. **a** Top mutated genes in 100 cSCC. Ranking is limited to genes with expression in keratinocytes and mutation frequency is shown in parentheses next to each gene name. **b** Analysis of the genes in **a** by mutation type. **c** Mutation frequency in *COL11A1* coding sequence. The number of mutations per amino acid (AA) is shown. **d** Mutation frequency calculated from The Cancer Genome Atlas (TCGA) of collagen genes with selected frequently mutated cancer genes from the Catalog of Somatic Mutations in Cancer (COSMIC) shown for reference.

We analyzed whole exome data of 100 cSCCs with patient-matched skin as control, including 33 newly generated for this study (SI Appendix, Table S1) [22–25]. These 100 cSCCs displayed a transition-rich mutational profile consistent with ultraviolet light exposure (SI Appendix, Fig. S1a) and featured mutations in genes that have previously been well-studied in cSCC, including *TP53* (78%), *NOTCH1* (59%), *CDKN2A* (41%), *KNSTRN* (17%), and *HRAS* (16%), at frequencies comparable to those seen in prior work (Fig. 1a and SI Appendix, Table S2) [21–26]. *COL11A1* was the third most commonly mutated gene (66%) after *TP53* and *CDKN2A* upon normalizing for coding sequence transcript length. Analysis of mutation type confirmed that while canonical tumor suppressors such as *TP53*, *CDKN2A*, and *NOTCH1* frequently harbor premature stop codons, cSCC-associated somatic variants in *COL11A1* were primarily missense mutations, suggesting functional consequences at the protein level that might be more nuanced than simple loss-of-function (Fig. 1b). The nature of *COL11A1* mutations was similar across cSCC datasets examined. In total, 61% (110 of 180) of these mutations affected glycine and proline residues, notably within Gly-X-Y triple helical repeats. We confirmed *COL11A1* mutations by Sanger sequencing (SI Appendix, Fig. S1b, c) and detected COL11A1 protein in every cSCC from an independent series (*n* = 76) of human tumors (SI Appendix, Fig. S1d, e), verifying its presence in this cancer. The predominance of missense glycine and proline substitutions (83%, 55 of 66 cSCC) concentrated in the triple helical region (Fig. 1c) indicated that these mutations might alter Gly-X-Y (with X and Y frequently proline) collagenous tripeptide [27] repeats, a phenomenon well-characterized for *COL7A1* [28–30] that disrupts the collagen triple helix and produces a dominant-negative collagen protein. In total, 42% (59 of 139) mutations detected in the *COL11A1* triple helical region were glycine substitutions, and 27% (38 of 139) altered a proline. Although their cancer incidence is unreported, the very rare autosomal dominant connective tissue disorders, Stickler and Marshall syndromes, can be caused by similar mutant *COL11A1* glycine substitutions in the Gly-X-Y tripeptide [31, 32], indicating that such mutations can act dominantly. *COL11A1* is thus the third most commonly mutated gene in cSCC, with a mutation pattern suggesting potential trans-dominant function.

To determine if *COL11A1* mutation is unique to cSCC, additional tumor types were examined. After assessment for background mutation rate differences in each tumor type, analysis of cancer sequencing data from TCGA found *COL11A1* commonly mutated across multiple epithelial malignancies, including cancers of the lung, esophagus, stomach, cervix, and colon (Fig. 1d). Mutations in other collagens, such as *COL6A6*, *COL22A1*, *COL6A3*, *COL12A1*, and *COL14A1*, were also noted in a variety of tumor types. Cancers displaying the highest frequency of collagen gene family mutations independent of *COL11A1* included cutaneous, gastrointestinal (esophageal, colorectal), urogenital (cervical, bladder), and lung neoplasms. Biologic malignancy in each of these tissues requires epithelial cells to invade through the epithelial basement membrane to penetrate into the underlying stroma. Collagen mutations are therefore a common feature of epithelial cancers.

### Mutant *COL11A1* promotes tumorigenesis

**Fig. 2:**
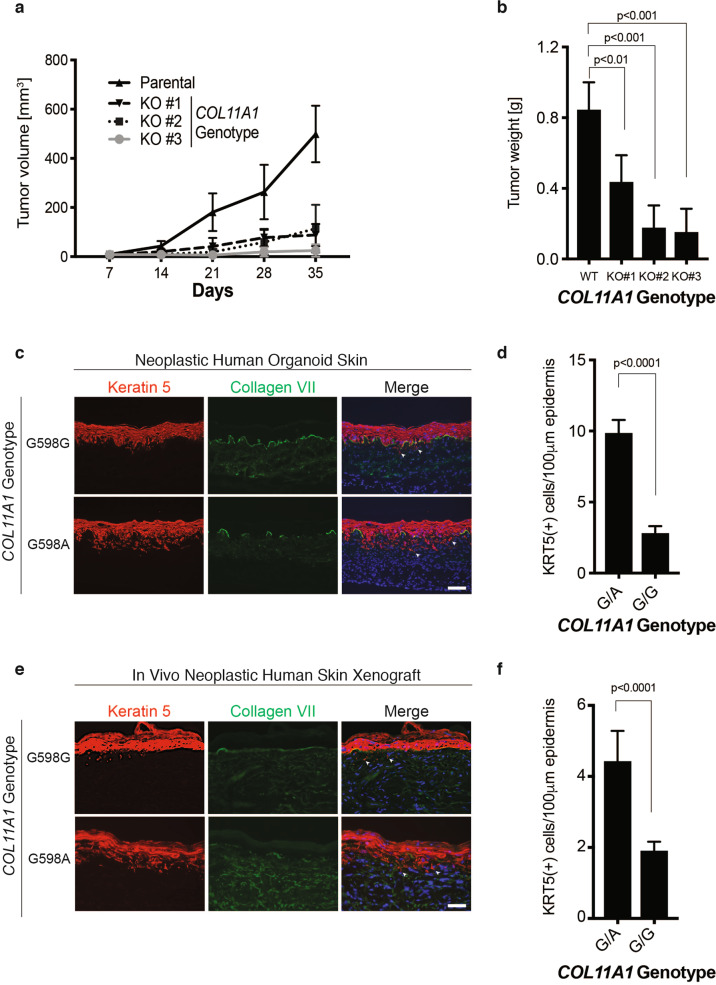
Mutant COL11A1 promotes tumorigenesis. **a** Tumor volume of A431 cSCC subcutaneous mouse xenografts. Parental A431 cells (WT) are compared to three independently derived *COL11A1* knockout clones (KO). **b** Tumor weight 35 days post-injection. **c** Isogenic, otherwise genetically identical human skin organoids programmed for invasive neoplasia by HRAS^G12V^ and Cdk4 with endogenous *COL11A1* edited to either cSCC-associated G598A or synonymous G598G wild-type control. Scale bar, 50 µm; arrowheads denote invasive cells, collagen VII staining defines the epidermal basement membrane. **d** Invasion index (number of keratin-positive cells/100 µm of basement membrane) of **c**; G/A, *COL11A1*^G598A^ and G/G, *COL11A1*^G598G^. **e** Parallel experiment from **c** xenografted onto *scid/scid* mice. Scale bar, 50 µm. **f** Invasion index of **d**.

To investigate the functional consequences of mutant *COL11A1* in cSCC, we established subcutaneous tumors in immune deficient mice using human A431 cSCC cells, which contain a G598A mutation in *COL11A1* representative of the glycine substitutions observed in cSCCs; A431 cells produce rapidly growing intradermal and subcutaneous tumors in this setting [33, 34]. CRISPR-mediated ablation of *COL11A1* in three independently derived A431 clones was associated with markedly impaired tumorigenic growth in vivo (Fig. 2a, b and SI Appendix, Fig. S2a–d). *COL11A1* knockout A431 tumors displayed evidence of modestly decreased proliferation in vivo (SI Appendix, Fig. S2e). These mutant *COL11A1* knockout data support the possibility that mutated *COL11A1* may contribute to tumorigenesis.

Epithelial cancers, including cSCC, arise via neoplastic invasion of epithelial cells through the underlying basement membrane. This process of tumorigenic progression is bypassed by tissue injection of cancer cell lines. Therefore, the impact of mutant *COL11A1* was next assessed in a more veridical model free of cell line-specific idiosyncrasies. A human skin tissue model of Ras-driven epidermal neoplasia, in which normal epidermis undergoes in situ neoplasia followed by progression to full invasion through the epidermal basement membrane, was therefore used to address the role of mutant *COL11A1* in early malignant progression. In both organoids and skin xenografts, this model accurately recapitulates cSCC at the levels of histology, protein markers, and global gene expression [18–20]. In this model, expression of HRAS^G12V^ and Cdk4, which reflect Ras-MAP kinase activation [23–25, 35] as well as inactivation of the *CDKN2A*-mediated G1 restraints characteristic of cSCC [18–20, 24, 36, 37] (Fig. 1a and SI Appendix, Table S2), are sufficient to drive cancer progression by normal diploid epidermal keratinocytes.

To define the impact of mutant COL11A1 on neoplastic progression to local invasion in the context of three-dimensional human skin tissue, wild-type human keratinocytes were edited to introduce the cSCC-associated *COL11A1*^G598A^ mutation using a combined CRISPR-Cas9 and adeno-associated virus-driven homology directed recombination approach (SI Appendix, Fig. S3) [38–40]. In parallel, keratinocytes from the same parental cell pool were edited with an A to T substitution that preserves the native glycine residue while controlling for any non-specific effects of editing the *COL11A1* locus (*COL11A1*^G598G^). These cells were used to regenerate epidermis on normal human dermis, producing two sets of human tissues for skin organoids and xenografts that differ by only a single nucleotide at the endogenous *COL11A1* locus. In human skin organoids, *COL11A1* mutation was associated with quantitatively increased neoplastic invasion (Fig. 2c, d) through the epidermal basement membrane into underlying dermal stroma, compared to otherwise genetically identical, gene-edited wild-type *COL11A1* control tissue. This result was replicated in xenografted human skin tissue in vivo (Fig. 2e, f), further confirming that cancer-associated mutations in the endogenous *COL11A1* locus are associated with enhanced neoplastic invasion in organoid tissues and in vivo.

### Mutant *COL11A1* gene expression and survival

**Fig. 3:**
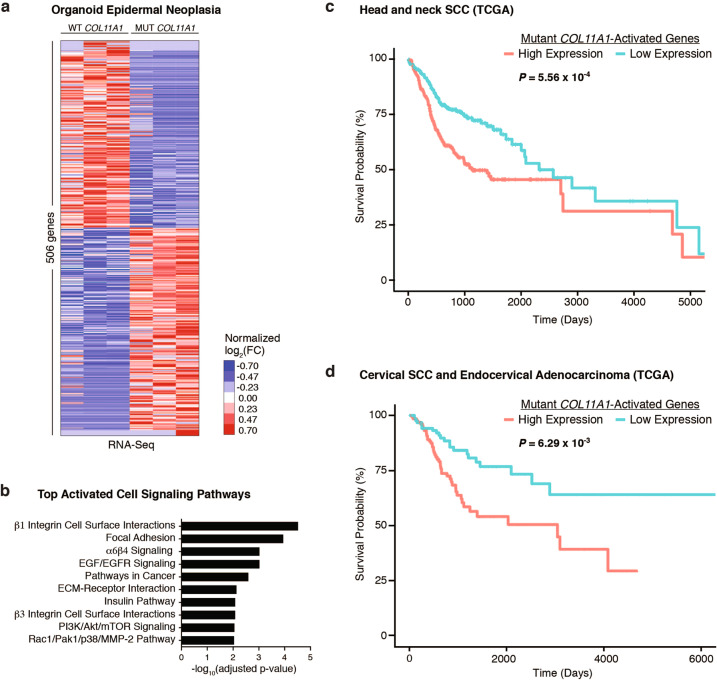
Genes induced by mutant *COL11A1* are associated with integrin signaling and worse survival. **a** Mean-centered, hierarchical clustering of 506 genes altered (adjusted *p* value <0.05) in isogenic human skin organoids with their endogenous *COL11A1* alleles edited to cSCC-associated G598A or synonymous G598G as control and programmed to transform into invasive neoplasia by oncogenic Ras and Cdk4. FC fold change. **b** Enriched GO terms of genes upregulated by *COL11A1*^G598A^ in RNA-Seq. **c**, **d** Kaplan–Meier estimate of overall survival using the mutant *COL11A1*-activated 264-gene signature in TCGA patients from the Head and Neck Squamous Cell Carcinoma (HNSC) and Cervical Squamous Cell Carcinoma and Endocervical Adenocarcinoma (CESC) cohorts, tumors chosen because, like cSCC, they comprise stratified epithelial cancers but which, unlike cSCC, have substantial sized cohorts of patient survival data.

To define the changes in gene expression induced by *COL11A1* point mutation in epidermis undergoing Ras-driven neoplasia, RNA expression was next compared in triplicate isogenic *COL11A1* mutant and triplicate wild-type tissues generated from identical donor cells that differ only in at the single-edited *COL11A1* nucleotide. Compared to control-edited *COL11A1* wild-type tissue, mRNA expression for 506 genes was significantly changed (increased or decreased) by *COL11A1* point mutation. The 264 upregulated genes were enriched for links to β1 integrin signaling, focal adhesion, and features of extracellular matrix-receptor interactions (Fig. 3a, b). We next evaluated the association between this 264-gene signature upregulated by mutant *COL11A1* and survival in head and neck SCC, which like cSCC is also a malignancy of stratified epithelium but which differs from cSCC in that it presently has long-term survival data available. Increased expression of the mutant *COL11A1*-activated gene signature was associated with decreased survival and demonstrated a 34% increase in hazard ratio (HR = 1.34, 95% CI = 1.14–1.59, *p* = 5.56e–4) and added 40% more information to a predictive model based solely on age, gender, clinical stage, and radiation therapy (Likelihood ratio test, *p* = 3.04e–4) (Fig. 3c and SI Appendix, Fig. S4a, b). High expression of the mutant *COL11A1*-activated gene signature was similarly associated with decreased survival in cervical cancer (HR = 1.41, 95% CI = 1.10–1.81, *p* = 6.29e–3) as well as lung SCC (HR = 1.16, 95% CI = 1.01–1.34, *p* = 3.79e−2), adding 27% and 26% more information, respectively, to a predictive model based on age, gender, clinical stage, and radiation therapy (Likelihood ratio test, *p* = 4.61e−3 and 3.66e−2) (Fig. 3d and SI Appendix, Fig. S4c–f). The gene signature upregulated by mutant *COL11A1* therefore correlates with decreased survival across multiple tumor types. Consistent with a potential link to integrin-driven gene expression, mutant *COL11A1* tissue displayed phosphorylated focal adhesion kinase, a downstream target of β1 integrin activation (SI Appendix, Fig. S4g). *COL11A1* point mutation therefore induces a gene set associated with overall worse cancer survival that is enriched for links to integrin signaling.

### Tissue mosaic for mutant *COL11A1*

The fact that collagens are secreted proteins, the resemblance of *COL11A1* tripeptide sequence mutants in cSCC to the dominant-negative secreted mutant COL7A1 protein seen in dominant dystrophic epidermolysis bullosa [41, 42], and the evidence above supporting engagement of integrin activation in tissue by mutant *COL11A1* all raised the possibility that mutant COL11A1 secreted from tumor cells might act on adjacent COL11A1 wild-type tumor cells. If so, cells expressing mutant COL11A1 protein should be able to enhance neoplastic invasion by adjacent *COL11A1* wild-type tumor cells when both contain oncogenic drivers.

**Fig. 4:**
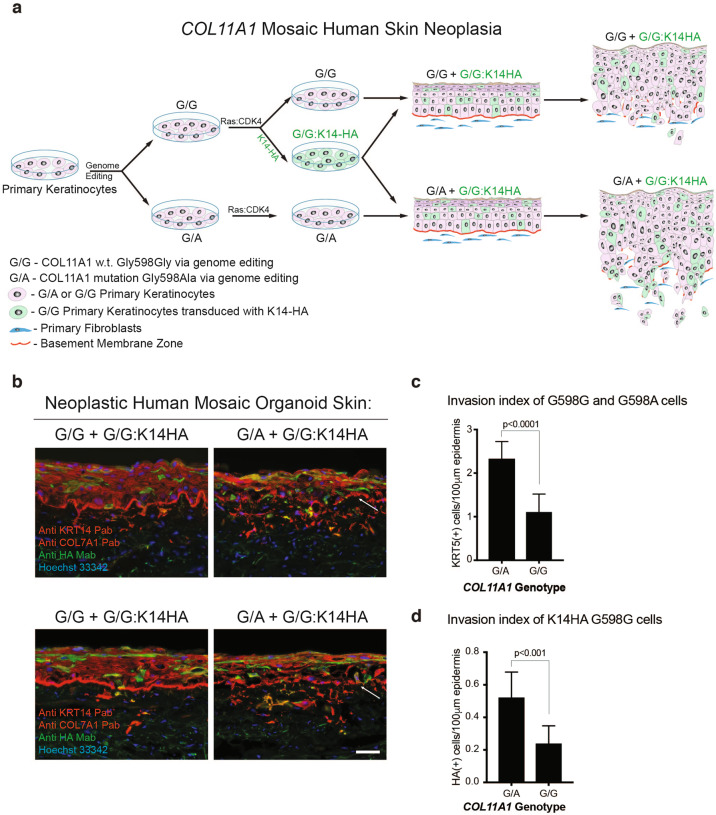
Tissue mosaic for mutant *COL11A1*. **a** Schematic illustrating generation of *COL11A1* mosaic human skin neoplasia. **b** HRAS^G12V^ and Cdk4 human skin tissues generated from keratinocytes with either endogenous *COL11A1* edited to cSCC-associated G598A or synonymous G598G as control; a subset of the latter group is further labeled with HA-epitope-tagged keratin 14 (G/G:K14-HA). Arrows point to the BMZ. Scale bar, 50 µm and technical replicates are shown. **c** Invasion index of *COL11A1*^G598A^ (G/A) and *COL11A1*^G598G^ (G/G) keratin 5-positive cells in **b**. **d** Invasion index of HA-tagged *COL11A1*^G598G^ (G/G: K14-HA) keratinocytes in *COL11A1*^G598A^ (G/A) and *COL11A1*^G598G^ (G/G) tissues in **b**.

To test this, mosaic human epidermis was generated in which all keratinocytes express HRAS^G12V^ as well as Cdk4 and were also edited to achieve either endogenous expression of mutant *COL11A1*^G598A^ or wild-type *COL11A1*^G598G^ as control (Fig. 4a). A subset of epidermal cells edited to wild-type *COL11A1*^G598G^ were marked with a hemagglutinin (HA)-tagged Keratin 14 expression vector and mixed at identical known percentages with unmarked *COL11A1*^G598A^ or *COL11A1*^G598G^ cells. Mosaic tissues with mutant *COL11A1* demonstrated quantitatively enhanced Ras-Cdk4-driven invasion over tissues mosaic for wild-type *COL11A1* (Fig. 4b, c) and the proportion of HA-tagged *COL11A1*^G598G^ cells crossing the epidermal basement membrane was significantly higher in mosaic tissues with mutant *COL11A1* compared to wild-type *COL11A1* mosaic tissues (Fig. 4d). The enhanced invasion by wild-type *COL11A1* cells adjacent to mutant *COL11A1* cells indicates that *COL11A1* mutations observed in cSCC can increase neoplastic invasion by adjacent *COL11A1* wild-type tumor cells.

## Discussion

Here we show that *COL11A1* is recurrently mutated in cSCC and that its cancer-associated mutation boosts neoplastic invasion in tissue. Mutant *COL11A1* enhanced invasion in both organoids and in vivo xenografts of human skin tissue and its targeted disruption blocked subcutaneous tumor growth as well. When compared to otherwise identical isogenic skin tissue, *COL11A1* point mutation engaged a gene expression program associated with β1 integrin activation. *COL11A1* was found to be mutated in additional epithelial cancers along with additional collagens, including *COL6A6*, *COL22A1*, *COL6A3*, *COL12A1*, and *COL14A1*. We did not observe a strong correlation between *COL11A1* mutations and mutations in *COL2A1*, *COL11A2*, *COL5A1*, or *COL5A2*, which encode distinct alpha chains that can form heterotrimers with collagen XI (SI Appendix, Fig. S5a, b). Similarly, examination of helix-disrupting somatic variants in the most frequently mutated collagen genes did not reveal strong mutational co-occurrence with *COL11A1* (SI Appendix, Fig. S5b). Mutant collagens include not just fibrillar collagens but also non-fibrillar basement membrane collagens, such as *COL4A4*, which was highly mutated across its multiple domains in cSCC (SI Appendix, Fig. S5c). These data suggest that mutations in a variety of collagen genes may play potential roles in cancer pathogenesis across diverse cancer types.

Acceleration of neoplastic invasion by mutant collagens may occur via a number of mechanisms. The trimeric structure of collagens make them susceptible to disruption by dominant-negative mutants, much like another structural family of multimers, the keratins [43]. Such mutants may disrupt structural integrity of fibrillar and non-fibrillar collagenous components of the very barriers they normally maintain. Additionally, and possibly as a consequence of such disruption of collagen multimers, possibly in a trans-dominant fashion for mutants such as G598A in COL11A1, mutant collagens may trigger receptors on cancer cells, such as integrins, which normally sense alterations in the extracellular environment in normal wound repair [44]. The precise factors promoting the increased invasion observed in mutant *COL11A1* tissue, however, are not defined and may include synergy with well-characterized secreted factors, such as TGFB. The high incidence of *COL11A1* mutations in the primary cSCC tumors studied here suggest that COL11A1 could act primarily in the process of local tumorigenesis as opposed to functioning as a strong driver of metastatic spread in this setting.

Based on similarities in gene expression programs, cancer has been compared to wounds that do not heal [45], suggesting that aberrant activation of wounding programs, such as those that drive cellular migration, may contribute to cancer progression. In the case of *COL11A1*, because it helps control fibril diameter of major stromal constituent collagens, such as type I collagens, its mutation in cSCC may disrupt the normal dermal collagenous matrix in such a fashion as to trigger such an aberrant wounding response. Assessment of collagen fibrils in *COL11A1* wild-type and mutant tissues stained with Masson’s trichrome to highlight collagens did not show quantitatively significant differences in our model cSCCs (Fig. S6A), however, more detailed ultrastructural studies are needed to definitively assess such potential impacts. Interestingly, *COL11A1* is not widely overexpressed in cSCCs, at either the mRNA [46] or protein levels, as shown here, raising the possibility that *COL11A1* mutations may in fact lead to a less stable protein, as is seen with other cancer mutant proteins. More generally, the common occurrence of collagen mutations in human cancer raises the possibility that these and other potential mechanisms operate via a variety of collagens in diverse epithelial malignancies.

Tumor cells interact with extracellular matrix proteins as they traverse both the basement membrane as well as its underlying stroma. Collagens comprise central proteinaceous components of both structures and are normally secreted into these locations by overlying epithelial cells as well as by stromal cells, most notably fibroblasts [47–53]. Interestingly, laser capture microdissection and sequencing of fibroblast regions of *COL11A1* mutant cSCC found only wild-type *COL11A1* sequence (Fig. S6B), consistent with a model in which epithelial cells serve as the source of mutant COL11A1 in cSCC. The present data thus support a model in which tumor cell-expressed mutant collagens boost a central process in cancer progression, namely invasion through the underlying epithelial basement membrane into the underlying stroma.

## Materials and methods

Please see additional methods in Supplementary Information.

### Tumor tissues

Cutaneous cSCC and patient-matched normal adjacent skin were collected following informed consent under a protocol approved by the Institutional Review Board at Stanford University. All tissues were analyzed by frozen section histology and samples with heavy neutrophilic infiltrate or widespread necrosis were excluded. Genomic DNA was isolated from all specimens using the DNeasy Blood and Tissue kit (Qiagen). Whole exome capture (Agilent SureSelect Human All Exon V5), library preparation, and sequencing were performed by Centrillion Technologies (Palo Alto, CA).

### Immunohistochemistry

COL11A1 immunohistochemistry (1:300, Biorbyt) was performed on a skin cancer and normal tissue microarray (Biomax) by the Stanford University Human Pathology/Histology Service Center. Ki-67 immunohistochemistry (1:200, Dako) was similarly performed on A431 xenograft tumors.

### RNA isolation and sequencing

Total RNA was then isolated from epidermis of the organoid skin samples using the QIAshredder (Qiagen) and Trizol (Thermo Fisher) followed by DNA removal with the TURBO DNA-free kit (Ambion) according to the manufacturer’s instructions. RNA integrity was verified using an Agilent 2100 Bioanalyzer. RNA-Seq libraries were prepared with the mRNA Seq Sample Prep Kit (Illumina, Inc.) as recommended by the manufacturer. Seventy-five bp paired-end sequencing reads were obtained using the Illumina HiSeq platform. DESeq was used to call differential gene expression with an adjusted *p* value cutoff of *p* < 0.05 [54].

### In vivo subcutaneous tumor formation assays

All experiments were performed with the approval of the Stanford University Administrative Panel on Laboratory Animal Care. In total, 1 × 10^6^ A431 clones engineered to contain deletion in *COL11A1* were suspended in a volume of 150 μl containing 50% Matrigel (BD biosciences) and injected with a 31 g needle into the subcutaneous space of immunodeficient 6–8 week old SHO female mice (Charles River). Tumor growth kinetics were monitored by caliper measurements weekly for 5 weeks. Tumor volume was calculated using formula *V* = 1/2(L × W × H), where L—length, W—width, and H—height of the tumor in mm. Tumors were explanted and weighed at the end of the experiment.

Each A431 clone was injected subcutaneously into a single mouse flank (*n* = 5 mice per clone). Parental A431 cells were injected subcutaneously into the contralateral flank (*n* = 15 mice). The sample size for these experiments was selected based on results from pilot studies and prior experience with tumor xenografts in mice. Sample exclusion criteria were preestablished and comprised of technical failures such as mis-injection of cells. Physical randomization was performed using animal tag number. Animal studies were not performed in a blinded fashion.

### In vivo skin xenograft model

For the composite skin graft production, neoplastic organoids consist of G/G or G/A keratinocytes were prepared as described above and at day 6 grafted on NOD SCID Gamma mice (Jackson) as previously described [55]. Briefly, 6–8 week old mice were anesthetized using isofluorane and after shaving the hair from the mouse flank, a rectangular region of mouse skin (~1.6 × 1.4 cm) was removed using a scalpel. A human skin organoid was then sutured to the mouse skin and dressed with the non-adherent dressing TELFA (Tyco Healthcare/Kendall) secured by Tegaderm (3M Health Care) and Coverlet adhesive dressing (BSN-JOBST). Finally, a double layer of Co-Flex (Andover) was wrapped around the mouse. The dressing was removed 9–12 days post-grafting and grafts were then further characterized. Five mice received *COL11A1*^G598A^ xenografts and five received *COL11A1*^G598G^ xenografts.

## Supplementary information

Supplemental Information

Table S1

Table S2

Supplementary Fig. S1

Supplementary Fig. S2

Supplementary Fig. S3

Supplementary Fig. S4

Supplementary Fig. S5

Supplementary Fig. S6
