# Supplementary material for: Mutant collagen COL11A1 enhances cancerous invasion
Source: Oncogene. 2021 Sep 28;40(44):6299–307. doi: 10.1038/s41388-021-02013-y (PMC8566234; doi:10.1038/s41388-021-02013-y)
Supplement: Supplementary file 1 — Supplemental Information [file 41388_2021_2013_MOESM1_ESM.docx]

**SUPPLEMENTAL INFORMATION**

Supplemental METHODS

*Somatic Mutation Analysis*

Somatic variants were analyzed from a total of 100 cSCC-normal pairs. For 53 cSCC-normal pairs, variants were called using an in-house somatic variant calling pipeline based on GATK Best Practices. The code for this pipeline is available at https://github.com/tjbencomo/col11a1-wes-pipeline. Briefly, reads were aligned to the reference genome using BWA-MEM^49^, marked for PCR duplicates using GATK MarkDuplicates^50^, and corrected for quality score errors using GATK BaseRecalibrator^50^. A Panel of Normals (PON) was then created using patient matched normal skin to filter out recurrent technical artifacts. Finally somatic variants were identified using the PON and Mutect2^51^. Variants were annotated by the Variant Effect Predictor software package^52^. Mutation calls from the remaining 47 cSCC-normal pairs were downloaded from their respective PubMed articles^23,25^. All mutations were mapped to the GRCh38 reference genome for analysis. The South et al. study displayed a higher incidence of transversions than the other datasets presented, possibly due to the fact that the primary cSCCs in that series are enriched for poorly differentiated cSCCs, although similar in body site, grade, and missense mutation impacts on *COL11A1* to the other studies.

*Cancer Mutation Frequency Analysis*

Mutation frequencies in Fig 1D were calculated by using available data from The Cancer Genome Atlas (TCGA). TCGA mutation and clinical annotations were downloaded through an R-API connected to the Cancer Genomics Data Center (https://cran.r-project.org/web/packages/cgdsr/, accessed May, 2020). All patients with mutation and Oncotree cancer type annotations were included. Mutation percentage was defined as the percentage of unique patients with a given cancer type with an exonic mutation in the indicated gene. Data obtained from TCGA were annotated with Oncotree codes and filtered to a select representative subset emphasizing cutaneous malignancies. Similar Oncotree codes were not merged and retain their original annotations. Background mutation burden was calculated for each cancer type by taking the mean of mutation percentages calculated individually for every gene (complete gene list obtained from https://www.genenames.org/). Known cancer genes were labeled by referencing the Catalog of Somatic Mutations in Cancer (COSMIC v91, released 07-APR-20). COSMIC genes displayed in Figure 1D were filter to those with greater than 5% mutation frequency in at least 7 displayed oncotree types. The analysis code can be accessed in the GitHub repository that accompanies this manuscript.

*Mutation Validation*

To validate selected somatic *COL11A1* variants, PCR primers that amplify the genomic region containing the mutation were designed. The amplification products were cleaned using the QIAquick PCR Purification Kit (Qiagen) and analyzed using Sanger sequencing. For each variant studied, the target region in both tumor and normal tissues was PCR-amplified and forward as well as reverse reads were generated. Sequencing chromatograms were then visually examined for evidence of the mutant allele.

| *COL11A1* Mutation | Forward Primer | Reverse Primer |
| --- | --- | --- |
| G74E | ggcaggagaatggtgtgaac | tgaatctgagagaatatttcgc |
| G736E | cccctttgaaggtgcatgt | accaatggctattaaaatatcaaagtg |
| G955E | caggctgtgaatttacctttg | ttgtaaataggtgggccattatc |
| G1003E | tggctaccaatgtggtttct | tttagaggtaatcacaattttgtttg |
| G1276R | aatttgcccaccaggaaata | tctttttctttcaaaacgtc |
| G1396D | tgctgtttttcagtattctaagagga | atggtggaacttaagggtgat |
| G1531E | caaaaacctcattgatattgtagca | tgacaaaaattcagatgcttca |
| P558S | ttggaatgtaatcaacattgag | gcagcttaactgggaatcgt |
| P858S | tcaatgcttccaggatttcag | gatgttcttgcatgactactc |
| P971T | gccctaccatgtgaaatattg | ctgactatgacaccatctact |
| P1206L/P1217S | tcacatgtggtcaacatttgc | gatttggagcaggtagagaat |

*Vector construction*

Dual gRNA expression lentiviral vector pZEX-2xU6COL11A1-CMV-PuroT2AmCherry was generated from pLEX-MCS lentivector with several modifications: The mammalian Hygromycin selection, bacterial Zeocin resistance cassette and IRES-Puromycin were removed and replaced by CMV-driven Puromycin in-frame fusion with T2A mCherry reporter gene. Upstream from expression cassette, human U6 promoter and F+E scaffold sequences were introduced separated by 600 nt stuffer to produce pZEX-GUIDE cloning vector. 170 nt Oligonucleotide containing 3’ end of F+E scaffold (34 nt), gRNA-R, 3’ end of human U6 promoter (15 bp), BsmBI flanked stuffer (36 nt), 3’ end of second F+E scaffold (15 nt), gRNA-F and 3’ end of second U6 promoter (33 nt) was synthesized at IDT. 1 ng oligonucleotide was amplified with high fidelity PrimeStar polymerase (Clontech) using 5’ oligonucleotide that restores full-length of F+E scaffold flanked with 15 nt synthetic sequence and 3’ double stranded DNA fragment containing second human U6 promoter to completion flanked with 42 nt synthetic DNA sequence. The 502 nt DNA fragment was re-amplified with 5’ biotinylated and 3’ phosphorylated primers and single stranded DNA was captured after denaturation and removal of the biotinylated strand using AmpureXP beads. This fragment was annealed to 54 nt RNA splint complementary to the flanking synthetic sequence and circularized in 10 μl reaction volume using SplintR ligase in the presence of 10 μM ATP for 1 hr at room T°C. The reaction volume was increased to 15 μl with Exonuclease I buffer and treated with 10 U Exonuclease I and 50 U exonuclease III for 30 min at 37°C and heat inactivated at 80°C for 20 min. 8 ng of circular DNA was used in 50 μl PCR reaction with inverse oligonucleotides targeting within the BsmBI flanked stuffer to produce final DNA fragment ready for in-fusion cloning into BsmBI digested pZEX-GUIDE plasmid to produce pZEX-2xU6COL11A1-CMV-PuroT2AmCherry vector. The reporter lentivector pLEX-K14HA was produced by PCR amplification of the human Keratin 14 cDNA (a gift from Dr. Todd Ridky) with primers allowing introduction of the 3xHA tag separated with Gly and Gly-Ser residues between each HA sequences at c-terminus of K14 gene. The final fragment was cloned in BamHI, XhoI digested pLEX-MCS plasmid to produce lentiviral K14-HA expression construct pLEX-K14HA-IRES-Puro. In order to construct the donor ssAAV vector used for homologous directed recombination (HDR), 1200 bp of the DNA sequence flanking the 5’ and 3’ end of the exon containing *COL11A1^G598^* was amplified from the genome and infused to an expression cassette consisting of the mouse PGK promoter, Blasticidin resistance gene and SV40 polyA signal. During amplification, either G to C or A to T point mutations were engineered within the targeted *COL11A1* exon resulting in G598A (G/A) or synonymous G598G (G/G) substitutions. Additionally, within the *COL11A1* intron, the PAM sequence for the targeting gRNA was mutated from AGG to AGC to prevent CRISPR/Cas9 mediated re-cutting. The expression cassette was flanked with LoxP recombination sites and the 22 nt DupC primer sequence from *COL11A1* intron was placed between the 3’ end of SV40 and LoxP. This allowed us to simplify HDR event analysis using genomic PCR with DupC primer and OutC, primer targeted outside of the homology region. The expected amplification results: 1306 nt for the G/A or G/G vs 1660 nt fragment indicative of the wild type allele (**SI Appendix, Figure S4**).

DupC: 5’ CCCAATAACAAGGAAAGGTAGG

OutC: 5’ TGTTCTATGCTGATTTGGTTGAA

AAV-DJ serotyped donor ssAAV virus was produced by the Stanford Neuroscience Gene Vector and Virus Core at genomic titer of 4.4x10^13^ TU/mL and used for CRISPR/Cas9 HDR experiments at MOI 2.5x10^5^.

***Cell culture***

A431 human epidermoid carcinoma cells were obtained genotype-verified directly from ATCC, mycoplasma tested, then cultured in DMEM supplemented with 10% FBS. Wild type male keratinocyte cells were isolated from neonatal foreskin and cultured in keratinocyte growth medium contained 50% Medium 154 (ThermoFisher) supplemented with HKGS (0.2% Bovine pituitary extract, (BPE), 5 µg/ml bovine insulin, 0.18 µg/ml hydrocortisone, 5 µg/ml bovine transferrin, 0.2 ng/ml human epidermal growth factor) and, 50% Keratinocyte SFM (KSFM, ThermoFisher) with supplemented with 0.1-0.2 ng/ml recombinant human EGF 1-53, 20-30 μg/ml, BPE. To separate the epidermis from the dermis, each skin sample was cut into four pieces and placed in 10ml PBS containing 25 caseinolytic units/ml of dispase (BD Biosciences) for 12 hours at 4°C. The next day, epidermis was carefully peeled off the dermis and placed in trypsin-EDTA (ThermoFisher) solution at 37°C for 15-20 minutes. The trypsin was quenched by adding equal volumes of a DMEM/10% FBS and then spun down at 1000 g to pellet the keratinocytes. Cells were washed once with PBS and plated on 10 cm dishes in keratinocyte growth medium. After 4-6 days, or when keratinocytes reached 60-70% confluence, cells were trypsinized and plated in 6-well plates for CRISPR/Cas9 and AAV-mediated HDR.

***CRISPR/Cas9-mediated knockout***

Human epidermoid carcinoma cells A431 were infected with lentivirus containing spCas9 at MOI 5 and seeded in a well of a 6-well plate. 48 hrs post-infection, cells were selected in 1.5 µg/mL Blasticidin for 72 hrs and transduced with dual sgRNA expression pZEX-2xU6COL11A1-CMV-PuroT2AmCherry lentivirus containing gRNAs (Forward and Reverse) flanking genomic locus encoded *COL11A1* signal peptide.

gRNA-F: 5’ gTTCCCCCTCTCCCTCCCCAA

gRNA-R: 5’ gCGCAGAAGCAGTAGGACCGA

Cells were cultured for two days, selected in 1 µg/mL Puromycin for 48 hrs and seeded in five 96 well plate with serial dilution. Wells containing single cell-derived populations were marked and collected by trypsinization after reaching 80% confluency and propagated further for genomic DNA isolation and *COL11A1* signal peptide deletion analyses using primers C11SP-F/R (**Supplementary** **Figure 3**).

C11SP-F: GCCCCTCATCTGCCTTCATT

C11SP-R: 5’ CAGATGTGTTCCAAGGGCAGT

***CRISPR/Cas9 and AAV mediated HDR***

The guide sequence targeting *COL11A1* for CRISPR/Cas9 genome editing was predicted using the CHOPCHOP web tool^53^. sgRNAs were ordered from Synthego and evaluated for genome editing efficiency in primary keratinocytes. The gRNA with highest editing efficiency was chosen for further use.

gRNA: 5’ gTTACCTGGAGCTAATGGTTA

180 pmol of the sgRNA was complexed with 20 pmol Recombinant 2xNLS-spCas9 protein (Synthego) in 30 µl of Amaxa nucleofection buffer for keratinocytes (Lonza) for 10 min and immediately used to nucleofect 2x10^5^ primary keratinocytes resuspended in 70 µl of nucleofection buffer with the Amaxa nucleofection apparatus (Lonza) using program T-018. After recovery cells were mixed with AAV-COL11A1^G598A^ or AAV-COL11A1^G598G^ at MOI 2.5x10^5^, split into 2 wells of a 6-well plate and propagated for 48 hrs following selection in 3 µg/mL Blasticidin for five days. During propagation, genomic DNA was isolated and evaluated for the editing efficiency using genomic PCR with primers DupC and OutC. The population of cells with at least over 30% editing efficiency were used for further experiments.

***Survival Analysis***

The association between the COL11A1-activated gene signature and survival was evaluated using data from the TCGA Head and Neck Squamous Cell Carcinoma (HNSC), Cervical Squamous Cell Carcinoma and Endocervical Adenocarcinoma (CESC), and Lung Squamous Cell Carcinoma (LUSC) cohorts. Clinical phenotypes and mRNA expression data was downloaded from the UCSC Xena database (https://xena.ucsc.edu/). Patients with complete information for mRNA expression, age, gender, clinical/pathologic stage, and radiation therapy were included in the analysis (n=436). A stratified Cox Proportional Hazards model with age, gender, clinical stage, radiation therapy, and the COL11A1-activated gene signature as covariates was fit to assess the relationship between the gene signature and survival. The model was stratified by radiation therapy (received treatment/didn’t receive treatment) as there was evidence that the proportional hazards assumption for radiation therapy was otherwise strongly violated. The model was fit using R and the rms package. The analysis code can be accessed in the GitHub repository that accompanies this manuscript.

***Statistical Analysis***

Statistical analyses were performed using GraphPad Prism software. Data are presented as mean ± SD. The statistical significance of differences observed in subcutaneous tumor growth studies and invasion index was determined using an unpaired two-tailed t-test assuming a Gaussian distribution.

***Neoplastic human organoid skin model***

The neoplastic human organoid skin culture was performed as described^19^. Briefly, devitalized human acellular dermis was used as the supporting framework for 3D cultures. Male human fibroblasts were introduced into the stromal side of the tissue and grown for at least 3 days in the dermis. COL11A1 genome edited keratinocytes were seeded onto the basement membrane side. Before seeding COL11A1^G598A^ (G/A) or COL11A1^G598G^ (G/G) cells were sequentially transduced with amphotropic retroviruses driving expression of CDK4^R24C^ and HRAS^G12V^. The organoid tissue was maintained in dual chamber supports separating the epithelial and stromal compartments for 8 days prior harvesting with media changes every day. For mosaic organoid skin culture G/G cells were also transduced with pLEX-K14HA-IRES-Puro lentivirus at MOI 3 and selected in 1µg/mL puromycin until control cells have fully cleared. Total of 0.5x10^5^ keratinocytes consist of 80% G/A and 20% G/G:K14-HA, or 80% G/G and 20% G/G:K14-HA and containing cancer associated genes, were seeded onto fibroblast impregnated dermis. Tissue was harvested in 8 days, embedded in OCT compound (Sakura) and subsequently cut into 10-µm cryosections throughout the sample. Every 10^th^ cryosection was collected, fixed in 50% acetone/50% methanol mixture for 10 min at −20°C and processed for immunofluorescence analyses according to standard techniques. Antibodies used: collagen VII (Santa Cruz, 1:50 dilution), keratin 5 (Covance, 1:600 dilution), keratin 14 (Covance, 1:600 dilution), HA (Abcam, 1:300 dilution), phospho-FAK Y397(R&D systems 1:10). Immunofluorescence images were taken using a Zeiss Axiovert inverted microscope and staining quantified using Fiji software. The invasion index was calculated by dividing amount of invasive keratin-positive cells in the dermis below the basement membrane/100um of basement membrane. At least 100 images per experimental group were analyzed.

Supplemental Figure legends

**Supplementary Fig. 1. *COL11A1* is somatically mutated in cSCC.** (**a**) Mutation types in 100 cSCCs. (**b**) *COL11A1* mutations confirmed as somatic by Sanger sequencing. (**c**) Representative chromatograms of *COL11A1* mutations present in cSCC. (**d**) Summary of collagen alpha-1 (XI) protein detection by immunohistochemistry in cSCC and normal skin microarrays. Tumors were blindly scored based upon strength of collagen alpha-1 (XI) stain. (**e**) Representative images of collagen alpha-1 (XI) staining in cSCC tissue. Scale bar, 50 μm.

**Supplementary Fig. 2. *COL11A1* deletion in A431 cSCC cells.** (**a**) Schematic illustrating CRISPR/Cas9-mediated deletion of the *COL11A1* signal peptide. gRNA, guide RNA. (**b**) Plasmid map of the targeting vector used to delete the *COL11A1* signal peptide in A431 cells. (**c**) Genomic analysis of A431 cells confirming deletion of the *COL11A1* signal peptide in 5 independently-derived clonal populations (KO) compared to the parental line (WT). (**d**) Clinical images of tumors from the mice in Fig. 2a-b. (**e**) Ki-67 immunohistochemistry of tumors from (d). Representative fields are shown (top). Scale bar, 25 μm. Ki-67 (+) cells in five 10X fields were quantitated (bottom).

**Supplementary Fig. 3. CRISPR/Cas9 and AAV-mediated base editing in primary keratinocytes.** (**a**) Schematic illustrating *COL11A1* point mutagenesis by CRISPR/Cas9 and AAV-mediated HDR (top). Sequencing chromatograms of *COL11A1* in wild type (WT) primary human keratinocytes edited to G598A or G598G. (**b**) Genomic analysis of Primary keratinocyte pools with their endogenous *COL11A1* alleles edited to G598A (G/A) or G598G (G/G). (**c**) Quantification of densitometry of (b).

**Supplementary Fig. 4. Impact of mutant *COL11A1* on survival and integrin action.** (**a, c, e**) Cox proportional hazard model of survival in the TCGA head and neck cSCC (HNSC), cervical SCC and endocervical adenocarcinoma (CESC), and lung SCC (LUSC) cohorts. HR, hazard ratio. CI, confidence intervals. (**b, d, f**) Cox multivariable analysis of the expression of the mutant *COL11A1*-activated 264-gene signature and survival in the TCGA HNSC, CESC, and LUSC cohorts after adjusting for age, gender (with the exception of CESC as all patients are female), clinical/pathologic stage, and radiation therapy. (**g**) Expression of phosphorylated FAK in human skin organoids with endogenous *COL11A1* edited to G598A that are programmed to undergo neoplastic transformation by HRAS^G12V^ and Cdk4. Arrow indicates malignant keratinocytes invading across the basement membrane. Scale bar, 50 μm. Human skin (hskin) control tissue is shown without primary antibody (-Pr Ab).

**Supplementary Fig. 5. Collagen gene mutation correlation.** (**a**) Co-occurrence of mutations in *COL11A1* and other collagen genes that encode components of the collagen XI heterotrimer. (**b**) The Jaccard coefficient was used to determine the strength of correlation between mutations in *COL11A1* and other collagens that contribute alpha chains to the collagen XI heterotrimer (bolded) as well as the most frequently mutated collagen genes. (**c**) cSCC mutation frequency in the *COL4A4* coding sequence. The number of mutations per amino acid (AA) is shown.

**Supplementary Fig. 6. Collagen fibril thickness and sequencing of laser capture microdissected human cSCC tissue**. (**a**) Collagen fibril thickness measured in isogenic human skin organoid tissues (n=2 each) gene edited for either mutant *COL11A1*^G598A^ or for *COL11A1*^G598A^ wild-type control stained with Masson’s trichrome; data represent 10 measurements per genotype and are presented as a percentage of average wild-type thickness+S.D. (**b**) Laser capture microdissection of spontaneous human *COL11A1* mutant cSCC. Left; histologic images at left (T=epithelial tumor, S=stroma) showing representative areas captured for sequencing. Right; *COL11A1* sequencing traces showing that heterozygous A/G nucleotide *COL11A1* mutation is only detected in tumor tissue as opposed to the wild-type G/G nucleotide in *COL11A1*, which was solely detected in adjacent stroma.

**Supplementary Table 1. Patient and tumor characteristics.**

**Supplementary Table 2. Full list of somatic mutations identified in 100 cSCC-normal pairs.**
