# Supplementary figures and images for: Mutant collagen COL11A1 enhances cancerous invasion
Source: Oncogene. 2021 Sep 28;40(44):6299–307. doi: 10.1038/s41388-021-02013-y (PMC8566234; doi:10.1038/s41388-021-02013-y)

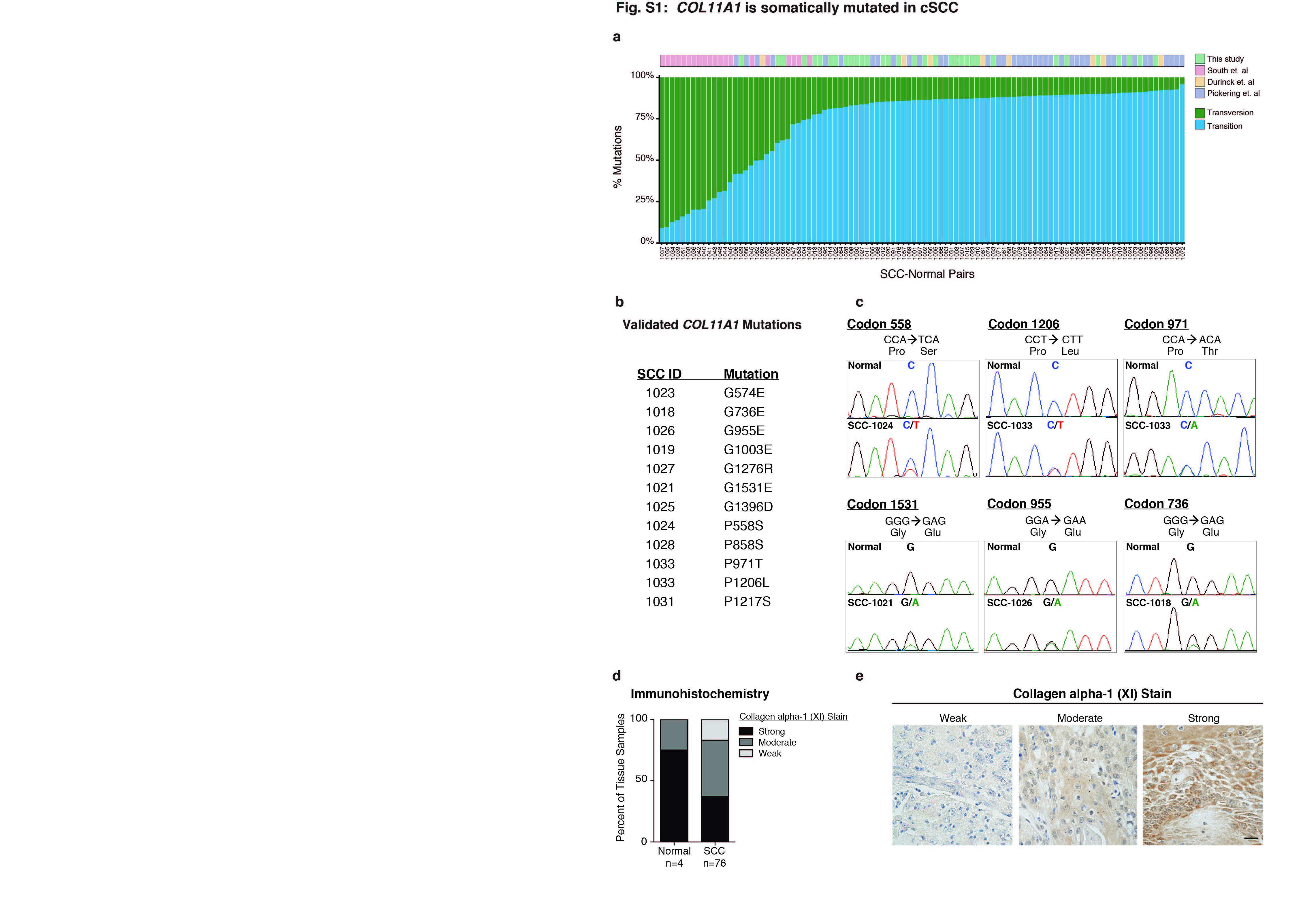

Supplement: Supplementary file 4 — Supplementary Fig. S1 [file 41388_2021_2013_MOESM4_ESM.jpg]

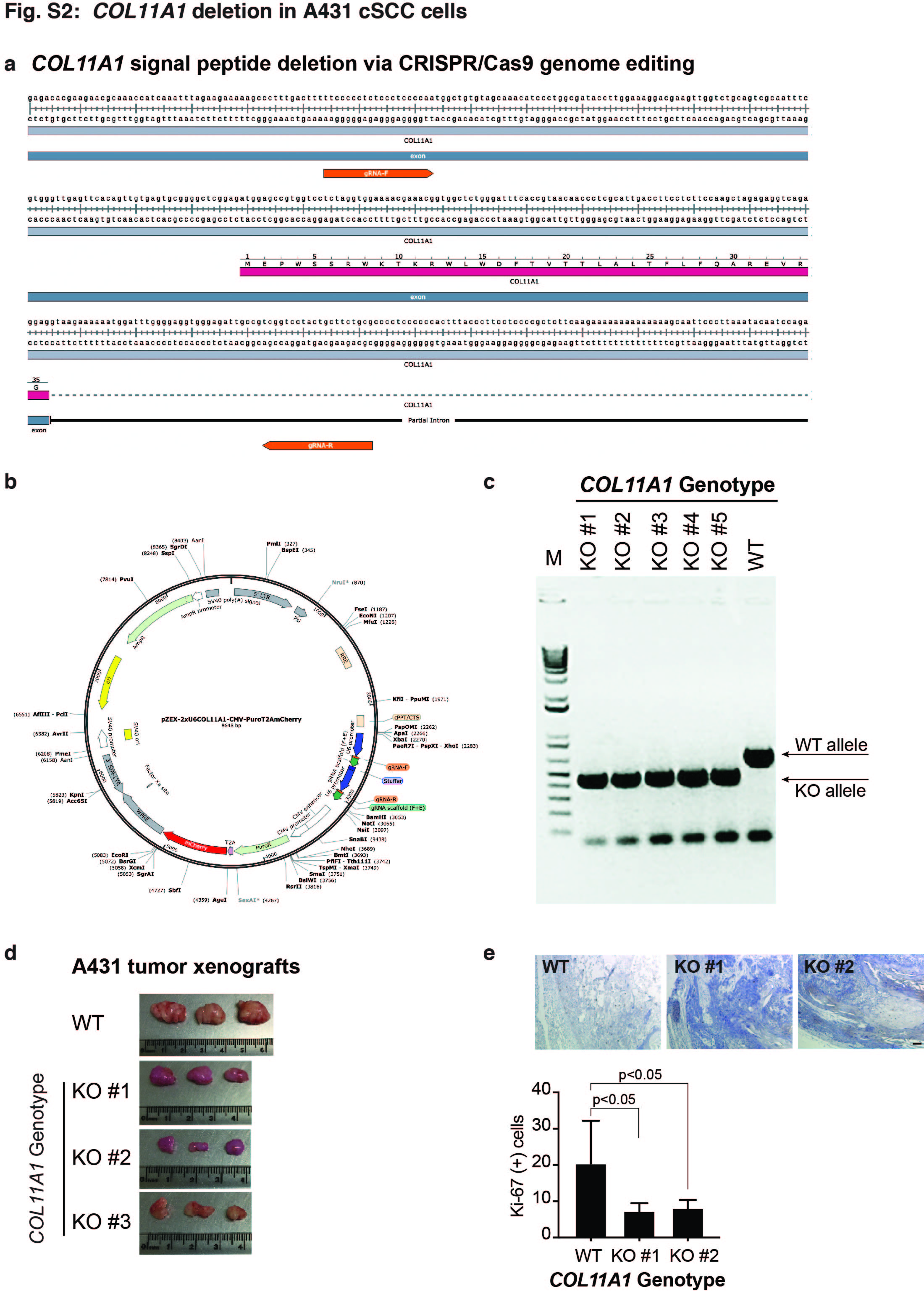

Supplement: Supplementary file 5 — Supplementary Fig. S2 [file 41388_2021_2013_MOESM5_ESM.jpg]

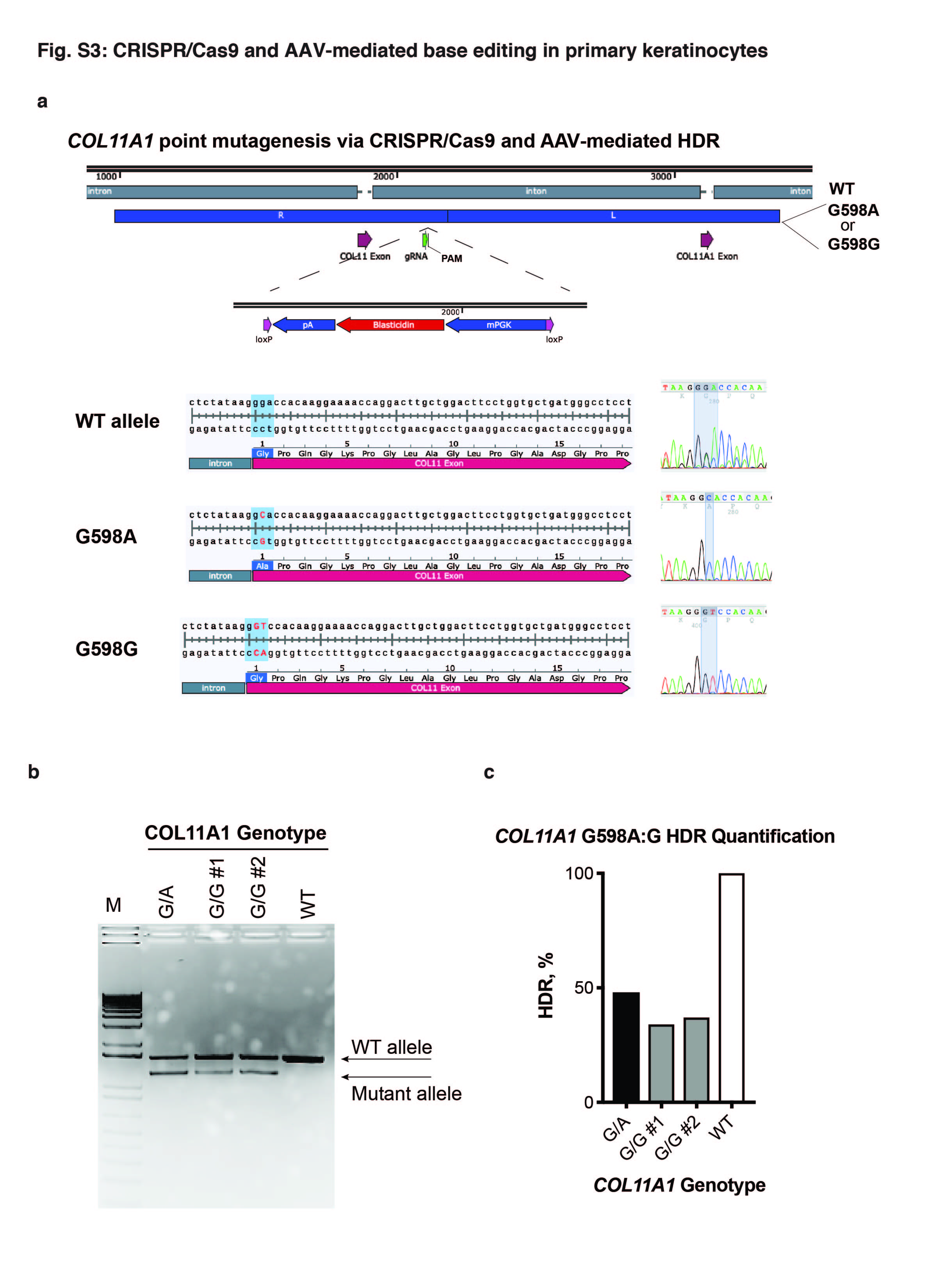

Supplement: Supplementary file 6 — Supplementary Fig. S3 [file 41388_2021_2013_MOESM6_ESM.jpg]

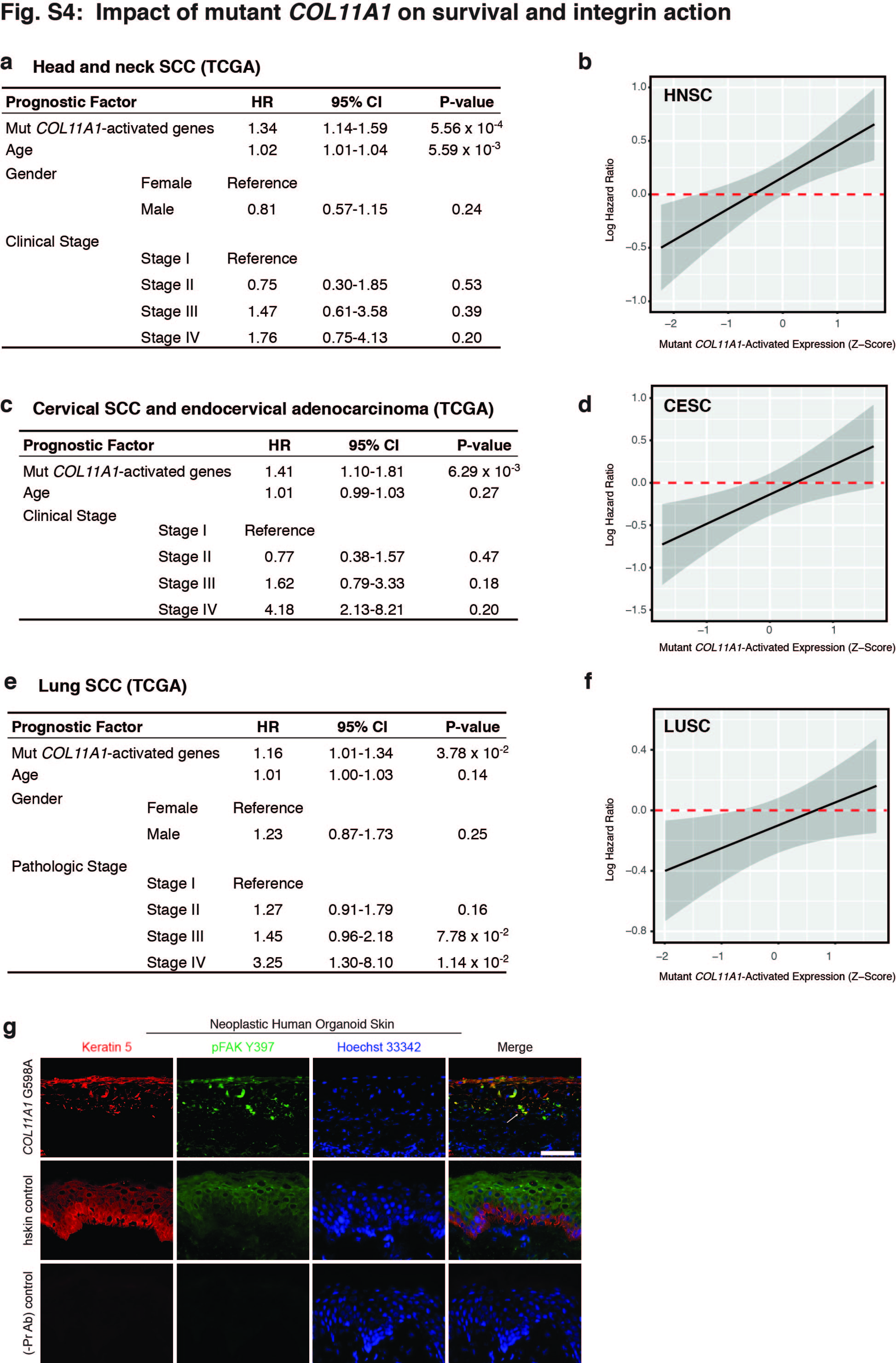

Supplement: Supplementary file 7 — Supplementary Fig. S4 [file 41388_2021_2013_MOESM7_ESM.jpg]

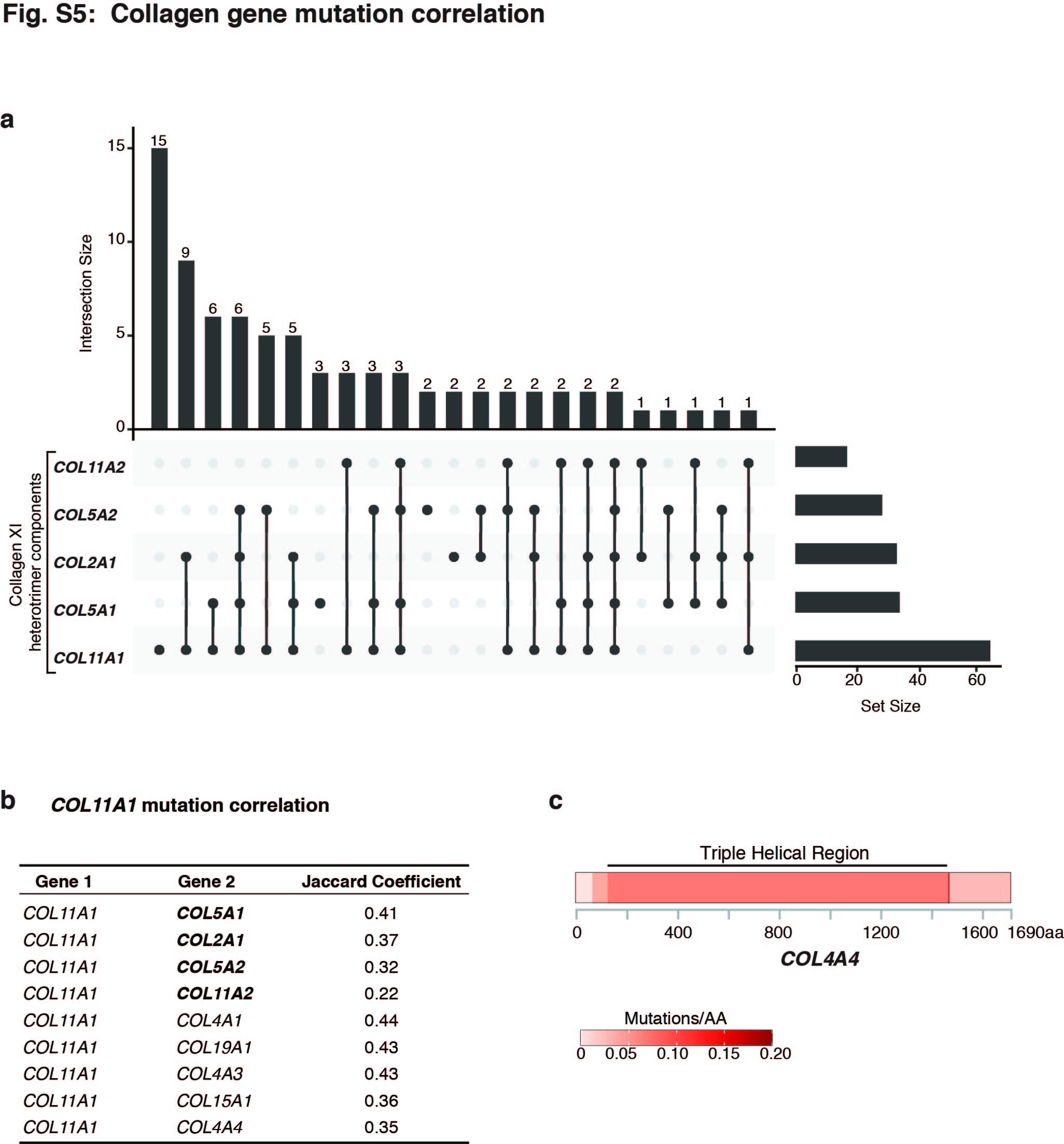

Supplement: Supplementary file 8 — Supplementary Fig. S5 [file 41388_2021_2013_MOESM8_ESM.jpg]

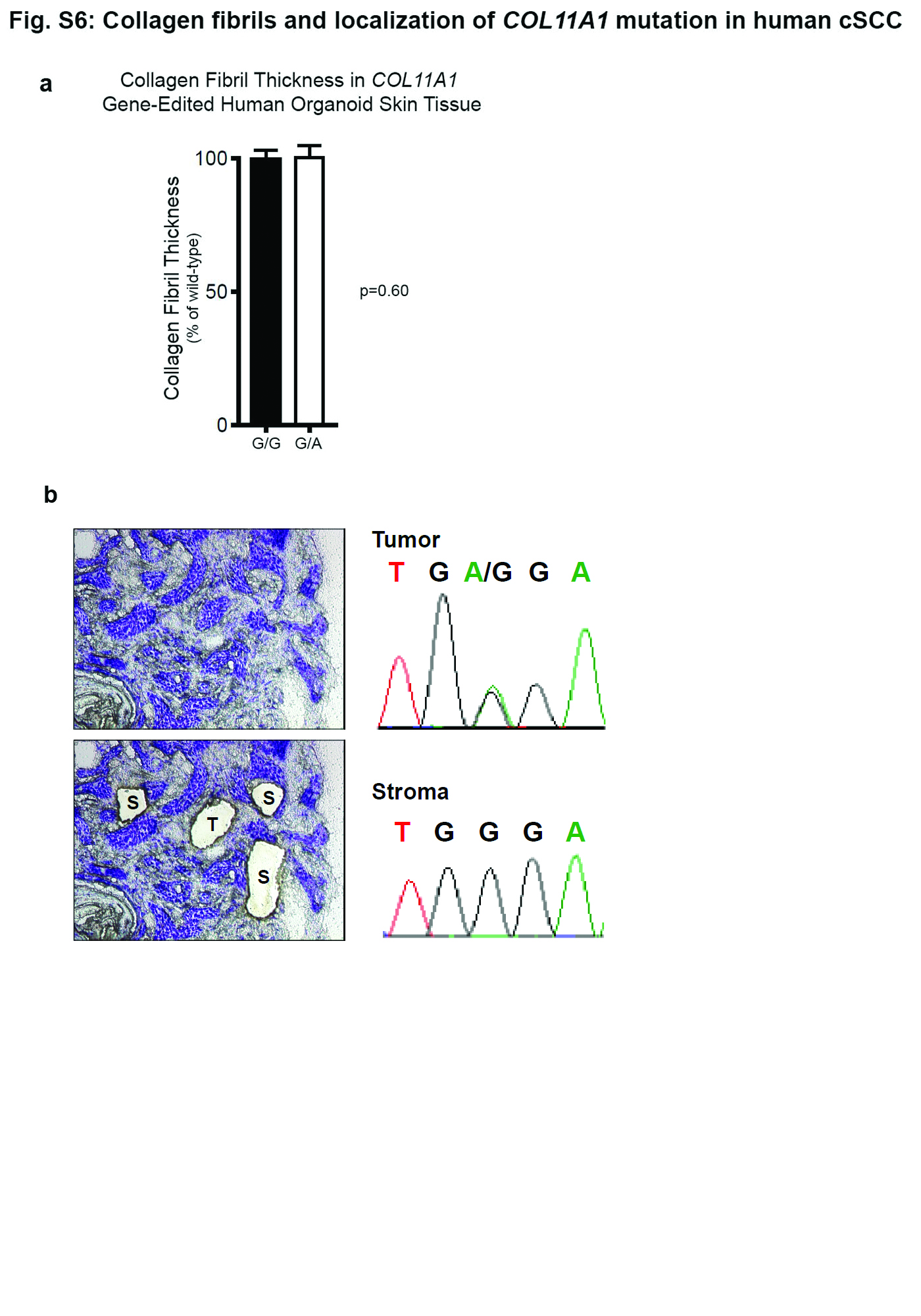

Supplement: Supplementary file 9 — Supplementary Fig. S6 [file 41388_2021_2013_MOESM9_ESM.jpg]
